# kalis: a modern implementation of the Li & Stephens model for local ancestry inference in R

**DOI:** 10.1186/s12859-024-05688-8

**Published:** 2024-02-28

**Authors:** Louis J. M. Aslett, Ryan R. Christ

**Affiliations:** 1 Department of Mathematical Sciences, Durham University, Stockton Road, Durham, DH1 3LE UK; 2 Department of Genetics, Yale School of Medicine, 333 Cedar Street, New Haven, CT 06520 USA

**Keywords:** Li & Stephens model, R package, Probabilistic haplotype model, Hidden Markov model, Genomics, High performance computation

## Abstract

**Background:**

Approximating the recent phylogeny of *N* phased haplotypes at a set of variants along the genome is a core problem in modern population genomics and central to performing genome-wide screens for association, selection, introgression, and other signals. The Li & Stephens (LS) model provides a simple yet powerful hidden Markov model for inferring the recent ancestry at a given variant, represented as an $N \times N$ distance matrix based on posterior decodings.

**Results:**

We provide a high-performance engine to make these posterior decodings readily accessible with minimal pre-processing via an easy to use package kalis, in the statistical programming language R. kalis enables investigators to rapidly resolve the ancestry at loci of interest and developers to build a range of variant-specific ancestral inference pipelines on top. kalis exploits both multi-core parallelism and modern CPU vector instruction sets to enable scaling to hundreds of thousands of genomes.

**Conclusions:**

The resulting distance matrices accessible via kalis enable local ancestry, selection, and association studies in modern large scale genomic datasets.

## Background

The hidden Markov model (HMM) of haplotype diversity proposed by Li & Stephens [1] (hereinafter, the LS model) has become the basis for several probabilistic phasing, ancestry inference, and demographic inference methods in modern genomics [2, 3].

Accelerated implementations of the LS model, typically targeting the Viterbi path, are integral to many commonly used genomics software packages, including BEAGLE [4], IMPUTE [5], and tsinfer [6]. A pioneering ancestry inference software package, ChromoPainter, popularized the idea of using the LS model to summarize the ancestry of $N$ haplotypes with an $N \times N$ similarity matrix [7]. This matrix is obtained by running $N$ independent HMMs in which each haplotype is modelled as a mosaic of all of the other haplotypes in the sample. This *‘all-vs-all’* copying approach is motivated by the product of approximate conditionals (PAC) likelihood originally proposed by [1] and allows ChromoPainter to render a chromosome-wide estimate of the recent ancestry of the $N$ haplotypes with high resolution.

The Relate [2] software suite extended this idea to performing local (locus-specific) ancestry inference along the genome. Internally, Relate uses high performance C++ implementations of the forward and backward algorithm to perform posterior decoding under a modified version of the LS model that incorporates derived allele information at many loci spaced along the genome. We will refer to this modified LS model as the derived allele haplotype copying model. These posterior decodings are transformed to $N \times N$ local genetic distance matrices and used to initialise variant-specific ancestral trees for downstream population genetic analyses ranging from demography to selection inference.

The current Relate software suite does not provide an interface for outputting the posterior decodings at a locus of interest and does not support the original LS model, only the derived allele haplotype copying model, which requires derived allele information.

Additionally, a LS-like model is implemented in [8] to run forward and backward recursions to variants of interest. However, the transition kernel used is different to the original LS model: upon a recombination event the transition kernel in [8] does not permit a haplotype to continue copying from the same donor haplotype.

The focus of kalis is to provide a high-performance engine to directly obtain the posterior decoding at a set of loci of interest for a dataset with hundreds of thousands of phased haplotypes. kalis supports the original LS model and the derived allele haplotype copying model. It provides a simple interface to enable rapid development of a range of future variant-specific ancestral inference pipelines on top, in the easy to use statistical programming language R [9].

At the same time, it has been recognised for over a decade [10] that the serial execution speed of CPUs will increase modestly, with additional performance primarily coming from concurrency via multi-core architectures or the growing width of specialised single instruction, multiple data (SIMD) instruction sets. Whilst multi-core architectures are now somewhat routinely exploited via forked processes or threading, SIMD instructions remain an often overlooked source of performance gains, possibly because they are harder to program. There are a cornucopia of SIMD instruction sets: on the Intel platform the genesis was in the 64-bit wide MMX instruction set [11] which allows simultaneous operation on two 32-bit, four 16-bit or eight 8-bit integers. The most recent incarnation on Intel CPUs is a suite of AVX-512 instruction sets [12], now capable of operating on 512-bits of various data types simultaneously (eg eight 64-bit floating point, or sixteen 32-bit integer values). Other CPU designs have similar SIMD technologies, such as NEON on ARM CPU [13] designs (including the Apple M1 and M2 processors, as well as Amazon Web Services Graviton range). Additionally all modern CPUs are superscalar architectures supporting instruction level parallelism, an advance that has been in the consumer Intel platform since the Pentium [14]. Judicious programming can make it easier for compilers and the deep reorder buffers of modern pipelined CPUs to exploit this more hidden form of parallelism.

In this work we provide a reformulation of the LS model and an optimised memory representation for haplotypes, which together enable us to leverage *both* multi-core and SIMD vector instruction parallelism to obtain local genetic distance matrices for problem sizes that previously appeared out of reach. This high performance implementation is programmed in C [15], with an easy to use interface provided in R [9]. We provide low-level targets of AVX2, AVX-512 and NEON instruction sets (covering the vast majority of CPUs in use today), and the whole package has an extensive suite of $162,835$ unit tests.

In the Implementation section below, we start with a description of the LS model and our reformulation which makes it amenable to these high-performance CPU technologies. We also describe the technical details of the underlying low-level implementation for the interested reader. We then demonstrate the performance that can be achieved with kalis, including examples with 100,000 haplotypes capable of running on a single machine. We also present a real data example using kalis to examine the ancestry at the *LCT* gene. In the following Discussion section, we describe the user friendly R interface which enables easy use of the high performance implementation without any knowledge of the underlying CPU technologies.

The kalis package is fully documented both within the package and on the package website https://kalis.louisaslett.com/.

## Implementation

### The LS model

To formalize our objective, let $h$ be an $L \times N$ matrix of $0$s and $1$s encoding $N$ phased haplotypes at $L$ sites. Let $h_i^{\ell } \in \{0,1\}$ denote the the $(\ell ,i)$th element of $h$. For brevity, let $h_i$ denote the $i$th haplotype (the $i$th column of $h$) and $h_{-i}$ denote all of the haplotypes excluding the $i$th haplotype. The LS model proposes an HMM for $h_i | h_{-i}$ in which the hidden state at variant $\ell$, $X^{\ell }_i \in \{1,\dots ,N\} \setminus i$, is an index indicating the haplotype in $h_{-i}$ that $h_i$ is most closely related to (or “copies from”) at variant $l$. We present here their proposed emission and transition kernels (see Equation A1 and Equation A2 in [1]) with a simplified parametrisation that is similar, but not identical, to that used by ChromoPainter.

While the original LS model assumes that each haplotype has an equal *a priori* probability of copying from any other, following ChromoPainter, we define a left stochastic matrix of prior copying probabilities $\Pi \in {\mathbb {R}}^{N \times N}$ where $\Pi _{ji}$ is the prior probability that haplotype $j$ is copied by $i$ and, by convention, $\Pi _{ii} = 0$. In other words, the donor haplotype (hidden state) that is sampled at the first variant and after every “recombination event” in the copying path is drawn according to $\Pi$. Here and whenever possible in kalis, all matrices are column-oriented such that the $i$th column pertains to an independent HMM where $h_i$ is treated as the observation. There is some probability of a mis-copy at variant $\ell$, $\mu ^{\ell }$, which under the LS model is set proportional to the mutation rate at $\ell$. This leads to an emission kernel of the form

1 $$\begin{aligned} \theta _{ji}^{\ell } := {\mathbb {P}}\left( h_{i}^\ell \left| X_{i}^{\ell } = j \right. \right) = {\left\{ \begin{array}{ll} 1 - \mu ^{\ell } &{} \text {if } h_{i}^\ell = h_j^\ell \\ \mu ^{\ell } &{} \text {if } h_{i}^\ell \ne h_j^\ell \\ \end{array}\right. } . \end{aligned}$$

The transition kernel between hidden states is based on the recombination rate between sites. Let $m^\ell$ be the genetic distance between variant $\ell$ and variant $\ell+1$ in Morgans (the expected number of recombination events per meiosis). Define $N_e = 4\tilde{N_e}/N$ where $\tilde{N_e}$ is the effective diploid population size (ie half of the haploid effective population size). Then, under the LS model the transition kernel is

2 $$\begin{aligned} \mathbb{P}(X_{i}^\ell = k | X_{i}^{\ell -1} = j) = \Pi _{ki} \rho ^{\ell -1} + {\textbf{1}}\left\{ k = j\right\} \left( 1-\rho ^{\ell -1}\right) , \end{aligned}$$

where $\rho ^\ell = 1-\exp \left( -N_e m^\ell \right)$ and ${\textbf{1}}\left\{ \cdot \right\}$ is the indicator function. Intuitively, this transition kernel asserts that upon a “recombination event,” where a recipient haplotype *i* may change the donor haplotype *j* it is copying from, the new donor haplotype is resampled from the prior copying distribution $\Pi _{\cdot i}$. [1, Appendix B] observe that in practice the estimation of recombination rates can be improved when the scaled recombination rate is raised to a power, so we adopt this approach and introduce an exponent $\gamma$. By default, kalis sets $\gamma = 1$, but this can be changed by the user. For $\gamma >1$ the recombination map becomes more heavily peaked, whereas $\gamma <1$ tempers the recombination map to make it more flat and smooth. Hence, in kalis, we set

3 $$\begin{aligned} \rho ^\ell := 1-\exp \left( -N_e \left( m^\ell \right) ^\gamma \right) , \end{aligned}$$

calculated using expm1() to help avoid underflow.

In keeping with the nomenclature introduced by [7], we refer to $h_i$ as the “recipient haplotype” and the remaining haplotypes, $h_{-i}$, as the “donor haplotypes”, in the context of the HMM where $h_{i}$ is treated as the emitted observation vector. This reflects the fact that each recipient haplotype $h_i$ is modelled as an imperfectly copied mosaic of the other observed haplotypes under the LS model. Hence, the posterior marginal probability at variant $\ell$, $p^{\ell }_{ji} := {\mathbb {P}}\left( \left. X_i^\ell = j \right| h\right)$, is the probability that donor $j$ is copied by recipient $i$ at variant $\ell$ given the haplotypes $h$. Under the above definitions of the prior copying probabilities $\Pi$, the emission kernel (1), and the transition kernel (2), the full $N \times N$ matrix of copying probabilities at $\ell$, $p^\ell$, can be obtained by running the standard forward and backward recursions [16] for each column (ie for each independent HMM).

From these posterior probabilities, we calculate a local $N \times N$ distance matrix, $d^\ell$. Firstly, notice that theoretically $p_{ij}^\ell > 0$, but it can be that $p_{ij}^\ell < \varepsilon$, where $\varepsilon$ is the double precision machine epsilon ($\approx 2.22\times 10^{-16}$, [15], pp.26). Effectively this means $d_{ij}^\ell$ is too large to reliably work with precisely, and so for the purposes of distance calculations we treat $\varepsilon$ as the smallest observable posterior probability, yielding

4 $$\begin{aligned} d_{ji}^\ell = -\frac{\log \left( p_{ji}^\ell \vee \varepsilon \right) + \log \left( p_{ij}^\ell \vee \varepsilon \right) }{2} \quad \forall \ j \ne i \end{aligned}$$

where $\vee$ is the maximum binary operator. By convention $d_{ii} = 0$ for all $i$.

We proceed in the next Section to reformulate the forward and backward recursions so that we can more fully exploit modern high-performance CPU instruction sets, while preserving numerical precision.

### Modification of the forward-backward algorithm

The $N$ independent HMMs of the LS model have forward and backward probabilities, respectively:

$$\begin{aligned} {\tilde{\alpha }}_{ji}^{\ell } = {\mathbb {P}}\left( X_i^\ell = j, h_i^{1:\ell } \right) , \qquad {\tilde{\beta }}_{ji}^{\ell } = {\mathbb {P}}\left( \left. h_i^{\ell +1 : L} \right| X_i^\ell = j \right) , \qquad i \in \{1,\dots ,N\} , \end{aligned}$$

where $h_{i}^{1:\ell }$ denotes haplotype $i$ from variant $1$ to $\ell$ inclusive.

Define,

5 $$\begin{aligned} F_i^\ell&:= \sum _{j=1}^N {\tilde{\alpha }}_{ji}^{\ell }& \quad \quad F_i^{0}&:= 1 \end{aligned}$$

6 $$\begin{aligned} G_i^\ell&:= \sum _{j=1}^N {\tilde{\beta }}_{ji}^{\ell +1}\theta _{ji}^{\ell +1} \Pi _{ji}&G_i^L&:= 1 \end{aligned}$$

Then the forward and backward recursions for the LS model can be written in vector notation (subscript $\cdot$ denoting a vectorised index),

7 $$\begin{aligned} {\tilde{\alpha }}_{\cdot i}^{\ell }&\leftarrow \theta ^{\ell }_{\cdot i} \left( \left( 1-\rho ^{\ell -1}\right) {\tilde{\alpha }}_{\cdot i}^{\ell -1} + \rho ^{\ell -1} F_i^{\ell -1} \Pi _{\cdot i} \right)&\text {for } \ell&\in \{2,\dots ,L\}, \end{aligned}$$

8 $$\begin{aligned} {\tilde{\beta }}_{\cdot i}^{\ell }&\leftarrow \left( 1- \rho ^{\ell }\right) {\tilde{\beta }}_{\cdot i}^{\ell +1} \theta ^{\ell +1}_{\cdot i} + \rho ^{\ell } G_i^\ell& \quad\quad\text {for } \ell&\in \{1,\dots ,L-1\} . \end{aligned}$$

with recursions initialised with $\alpha _{\cdot i}^{1} \leftarrow \theta ^{1}_{\cdot i} \Pi _{\cdot i}$ and $\beta _{\cdot i}^{L} \leftarrow 1$. Note that Eq. (7) corresponds to Equation A5 in [1].

To partially mitigate the risk of underflow, the forward recursion can be rearranged in terms of $\alpha _{\cdot i}^{\ell } := \frac{{\tilde{\alpha }}_{\cdot i}^{\ell }}{ F_i^{\ell -1}}$, and the backward recursion in terms of $\beta _{\cdot i}^{\ell } := \frac{{\tilde{\beta }}_{\cdot i}^{\ell }}{G_i^{\ell }}$ (see Additional file 1 for details). Thus, in full for $\ell \in \{1,\dots ,L\}$ we compute,

9 $$\begin{aligned} \alpha _{\cdot i}^{1}&\leftarrow \theta ^{1}_{\cdot i} \Pi _{\cdot i}&\quad\quad\quad\quad\text {for} \quad \ell = 1 \end{aligned}$$

10 $$\begin{aligned} \alpha _{\cdot i}^{\ell }&\leftarrow \theta ^{\ell }_{\cdot i} \left( \left( 1-\rho ^{\ell -1}\right) \frac{\alpha _{\cdot i}^{\ell -1}}{\underset{j}{\sum }\ \alpha _{ji}^{\ell -1}} + \rho ^{\ell -1} \Pi _{\cdot i} \right)&\text {for} \quad \ell > 1 \end{aligned}$$

and

11 $$\begin{aligned} \beta _{\cdot i}^{L}&\leftarrow 1&\quad\quad\quad\quad\quad\quad\text {for} \quad \ell = L \end{aligned}$$

12 $$\begin{aligned} \beta _{\cdot i}^{\ell }&\leftarrow \left( 1- \rho ^{\ell }\right) \frac{\beta _{\cdot i}^{\ell +1} \theta ^{\ell +1}_{\cdot i}}{\underset{j}{\sum }\ \beta _{ji}^{\ell +1}\theta _{ji}^{\ell +1} \Pi _{ji}} + \rho ^\ell&\text {for} \quad \ell < L \end{aligned}$$

Given $\alpha _{\cdot i}^\ell$ and $\beta _{\cdot i}^\ell$, the vector of posterior probabilities for recipient $i$, $p^\ell _{\cdot i}$, can be calculated directly by normalising,

13 $$\begin{aligned} p^\ell _{\cdot i} = \frac{\alpha ^\ell _{\cdot i} \odot \beta ^\ell _{\cdot i}}{\sum \limits _j \alpha ^\ell _{ji} \odot \beta ^\ell _{ji}} \end{aligned}$$

where $\odot$ denotes the Hadamard product. In the event that $\sum \limits _j \alpha ^\ell _{ji} \odot \beta ^\ell _{ji} = 0$, the distance between the recipient haplotype $i$ and all of the donor haplotypes is beyond numerical precision, so as per the earlier discussion we define $p_{ji}^\ell = \varepsilon \ \forall \ j \ne i$.

Finally, the local distances follow by taking the negative log and symmetrising. Note that if the distances are standardised for one of these columns, to account for the fact that the standard deviation will be 0, we set all of the standardised distances to 0. Please see Additional file 1 for a discussion on parameter values and exactly how kalis performs certain computations to maintain the numerical stability of the algorithm.

### Core implementation details

The R interface described hereinbefore is a thin wrapper layer around a high-performance implementation of the core algorithm which is written in standards compliant C18 [15]. Most data structures are represented with native R types enabling user inspection and manipulation, except for the haplotype sequences themselves.

Computationally, the innermost forward and backward recursions are implemented using compiler intrinsics to exploit a variety of modern CPU instruction sets, including Streaming SIMD Extensions (SSE2 and SSE4.1), Advanced Vector Extensions (AVX, AVX2, AVX-512 and FMA) and Bit Manipulation Instructions (BMI2) on Intel platforms; as well as NEON on ARM platforms. AVX2 is supported in Intel CPUs of the Haswell generation (released Q2 of 2013) or later, AVX-512 tends to be available only in recent Intel server and workstation grade CPUs, and NEON is available for ARM Cortex-A and Cortex-R series CPUs, as well as Apple M1/M2 and Amazon Web Services Graviton processors. Although this covers most CPUs likely to be in use today, we none-the-less provide reference implementations in pure standards compliant C which will operate on any CPU architecture with a C18 compliant compiler. During package compilation, the correct code-paths are compiled based on detection of the presence or absence of the required instruction sets, or at the direction of the user via compiler flags. See Additional file 1 for more details, and for guidance on how to directly check your CPU for SIMD support.

It may be worth noting at this juncture that it was an explicit design choice to target CPUs and not GPU or tensor cards initially. This is because most University high performance computing clusters have plentiful CPU resources, often with untapped power in advanced SIMD instructions sets. We believe that the problem size that can be realistically tackled in many genetics studies can be massively increased *without* needing to resort to add-on cards, though to scale beyond even this we may explore heterogeneous computing architectures in future kalis research.

In this section, we now describe the inner workings and design principles of the package, first covering in detail the data structures (both user facing and internal), followed by the computational implementation.

### Data structures

There are three user accessible data structures utilised in the package and a low level binary haplotype representation which is not directly user accessible. The two principle data structures of interest to users are forward and backward table objects, represented as native R lists with respective S3 class names kalisForwardTable and kalisBackwardTable (detailed in Table 2 and discussed later), which are created with package functions MakeForwardTable() and MakeBackwardTable() respectively. The third user accessible data structure holds the LS model parameters, represented as a native R environment with S3 class name kalisParameters, which can be created with the package function Parameters().

#### Haplotype data

The haplotypes are stored in an optimised binary representation which is only natively accessible from within C. Note that here “optimised” is not a reference to space-optimisation: it would be possible to represent the haplotypes in an even more compressed manner, but we aim for streaming compute speed optimisation instead.

The haplotypes are loaded from disk and transformed to an in memory cache in this representation via CacheHaplotypes(), but this function does not return any handle to the loaded data. Thus the package provides the accessor function QueryCache(), which copies genome segments from the binary representation into native R integer vectors for user inspection.

**Fig. 1:**
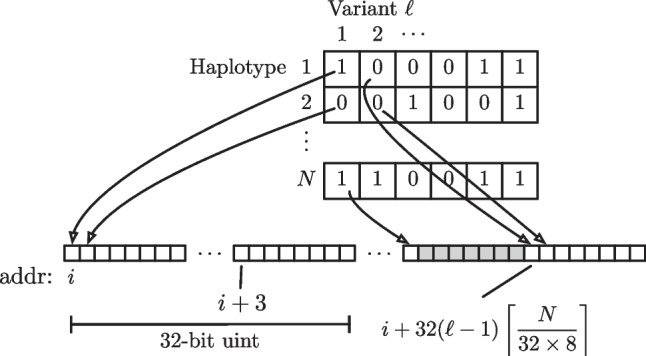
Efficient binary representation of interleaved haplotypes in memory, with 32-byte boundary alignment for each variant start for SSE/AVX instructions (here $i \mod 32 = 0$). The grey boxes indicate essentially ‘wasted’ bits which are ignored to ensure alignment for the start of the next variant

When CacheHaplotypes() loads haplotypes into the cache, they are interleaved into a flat memory space which is organised as variant-major. That is, variant 1 of each haplotype is loaded, converted to a binary 0/1 and then 32 consecutive haplotypes are packed into an unsigned integer. Moreover, the initial flat memory allocation is aligned on a 32-byte boundary to satisfy memory alignment requirements for some CPU vector instructions^1^, and after all haplotypes at a given variant are packed into consecutive unsigned integers the pointer is wound forward to the next 32-byte boundary to ensure the next variant starts on an SSE/AVX vector compatible memory boundary. This is depicted in Fig. 1.

Firstly, note that this orientation is natural, since the forward and backward recursions operate variant by variant, meaning variant-major storage ensures sequential memory locations are fetched during a recursion. Indeed, with the cache line size of 64-bytes (starting Intel Pentium IV), we essentially trigger the loading of $64 \times 8 = 512$ neighbouring variants upon accessing the first variant in a recursion. This effect is even more pronounced on Apple M1/M2 whose cache line size is 128-bytes, resulting in 1024 variants being pre-fetched upon access to the first variant in a recursion.

Secondly, a possible drawback is that we must extract the individual bit into a double floating point representation in order to compute with it in the recursion. However, efficient CPU instructions can help here too: take for example the following strategy kalis uses on an AVX2 capable CPU. Using the PDEP instruction in BMI2, we can efficiently deposit a bit into every ninth bit of an int (so there are now 4 8-bit integers taking on the value of the haplotype at this variant packed in an int). Then, using SSE2, SSE4.1 and AVX instructions one can inflate through representations from 4 8-bit integers packed in an int up to 4 64-bit doubles packed in a 256-bit AVX register. As such, we are then ready to operate with this variant in parallel using AVX instructions.

During development, testing indicated the memory bandwidth and cache efficiency savings of the packed binary representation provided speed-ups thanks to these instructions efficiently enabling unpacking and spreading a haplotype variant bit for parallel use. Furthermore, such a compact representation means that more of L1/L2 cache and memory bus bandwidth is left available for forward and backward tables, which are the largest objects we work with in this problem.

#### Parameters

The parameter set used by kalis can be created by calling the Parameters() function, which retuns a kalisParameters object with structure shown in Table 1. This structure corresponds to the parameters required to specify the LS model (Eqs. (1) and (2)). To calculate $\rho$ from a recombination map, $N_e$ and $\gamma$, we also provide a helper function, CalcRho(), which implements Eq. (3).

The kalisParameters object uses an environment rather than list for parameters for two reasons: (i) the parameter environment and its bindings are locked which prevents changes in parameter values between forward or backward table propagation steps, since parameters must be fixed for all steps of a given forward or backward computation; and (ii) an environment explicitly ensures the (often large) parameter vectors are not copied when associated with potentially many different tables, but will always be purely referenced.

The environment contains only two members: another environment with the actual parameter values (which is locked with lockEnvironment()); and a SHA-256 hash of those parameter values (details in Table 1). The purpose of the hash is to be able to efficiently determine whether the correct parameter set for a given forward or backward table has been passed when computing forward or backward recursions from an already initialised table (since it would be incorrect to propagate forward or backward using different parameter sets in different parts of the genome).

**Table 1:** The content of the data structure representing parameter objects

**kalisParameters** object	Data type	
pars	Locked R environment, containing:	
	rho	Vector length *L*
	mu	Vector length *L*, or scalar
	Pi	$N \times N$ matrix, or scalar
sha256	Character	

#### Forward/backward tables

**Table 2:** The content of the core data structures representing forward and backward table objects, together with their correspondence to mathematical quantities

**kalisForwardTable** object		kalisBackwardTable object		Data type
alpha	$= \alpha ^\ell _{\cdot \cdot }$	beta	$= \beta ^\ell _{\cdot \cdot }$	$N \times N$ matrix
alpha.f	$= F^\ell$	beta.g	$= G^\ell$	Vector length *N*
l	$= \ell$	l	$= \ell$	Integer scalar
from_recipient		from_recipient		Integer scalar
to_recipient		to_recipient		Integer scalar
pars.sha256		pars.sha256		Character
		beta.theta		Logical scalar

Recall that the recipients (columns) in the forward/backward tables correspond to independent HMMs. Therefore, kalis enables storing only a ‘slice’ of recipients in a forward/backward table, making parallelisation across non-shared memory clusters much simpler: given all haplotype data, these recipient slices can be independently propagated in a communication free manner.

The forward and backward table objects contain not only the (upto) $N$ independent forward/backward vectors at variant $\ell$, but also supporting meta-data. This includes the variant the table is currently at, the scaling constants $F^\ell$ (forward, Eq. (5)) or $G^\ell$ (backward, Eq. (6)), the range of recipient haplotypes represented (that is, the recipient HMMs to which the column corresponds), and a hash of the parameter values used in propagating this table.

In total, a full-size forward table for example requires $8N^2+8N+1576$ bytes of memory^2^ for storage and the small overhead of R object management. Since this grows quadratically in the number of haplotypes, most functions in the package operate on forward and backward table objects in-place, rather than via the idiomatic copy-on-write mechanism of standard R. The most important consequence of this for users is that standard assignment of a table object to another variable name only creates a reference and so an explicit copy must be made by using the CopyTable() utility function provided in the package.

### Core SIMD code

The two most important core algorithms which are accelerated with SIMD vector instructions are the forward and backward recursions. This code is fully implemented in C, with tailored modifications accounting for all combinations of: scalar/vector $\mu$, scalar/matrix $\Pi$, and use of the asymmetric mutation model of RELATE [2] or not (ie 8 combinations); to ensure that minimal memory accesses are performed where possible. So, for example, scalar $\mu$ and scalar $\Pi$ parameters will be faster than any other combination since these values are likely to be held in registers (or at least L1 cache) for the duration of the recursion.

Additionally, in all places where we identify SIMD instructions may be used, a macro is deployed, with a header file providing all mappings from these macros to a specific SIMD instruction for all supported instruction sets. Taking arguably the simplest non-trivial example, all src/ExactForward*.c and src/ExactBackward*.c files make us of the custom macro KALIS_MUL_DOUBLE(X, Y) when they need to multiply KALIS_DOUBLEVEC_SIZE double precision floating point values together. The file, src/StencilVec.h then provides definitions for these macros under each instruction set kalis supports (via assembly intrinsics), together with a pure C alternative. For this example, we have (with ... indicating other macro definitions):
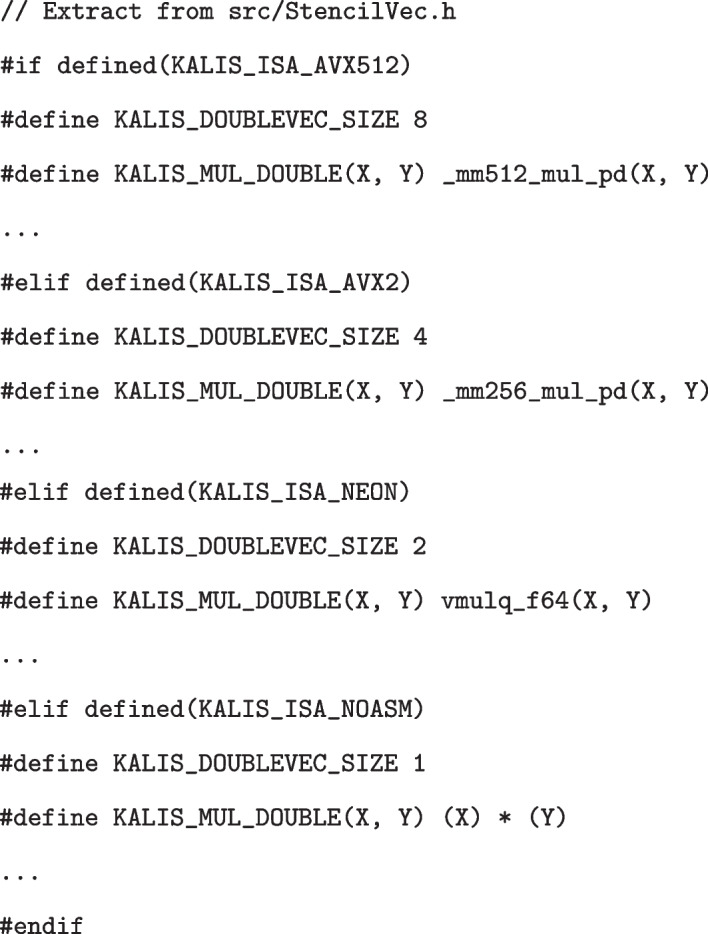


The inner-most loop in these core files then includes a programmatically generated unroll to the depth specified during compilation. All this is wrapped in code which dispatches using pthreads to multiple threads, with automatic detection of the ability to pin to specific cores if that option is passed (important in some settings to ensure a hot L1/L2 core cache). In particular, each thread operates on a subset of columns of the forward and backward tables, ensuring spatial locality for memory accesses. Furthermore, when propagating by more than a single variant position, each column (ie each independent HMM) is propagated all the way to the target variant before proceeding to the next column, ensuing temporal locality of memory accesses.

### Unit tests

Given the complexity of the development described above, we have implemented a comprehensive suite of unit tests to ensure correctness. Internal to the package is a “gold master” implementation of the LS model, which is a pure R implementation that has been written for correctness and is not optimised for speed. These pure R implementations are callable with an undocumented argument option to the standard Forward() and Backward() functions: if the argument nthreads = “R” rather than a numeric value, then the gold master implementation is used (at the cost of running significantly slower).

Unit tests fall broadly into two categories, one verifying the correctness of loading from the different supported input formats (via R matrix, .hap.gz and h5) into the optimised binary representation of Fig. 1, the other checking forward and backward computations against a ground truth computed by the gold standard R implementation. The latter category of tests are the most extensive, since they cover tests of all combinations of: single threaded and multi-threaded computation; moving different numbers of variants in a single call; different problem sizes where the numbers of haplotypes is either exactly divisible by the CPU vector unit length (i.e. 256-bits for AVX2 etc), or has different remainders; original LS and derived allele haplotype copying model; scalar and vector mutation probabilities ($\mu$); uniform and matrix copying probabilities ($\Pi$); and in the case of backward recursions, all combinations of starting and ending a recursion in standard or rescaled probability space (beta.theta argument to Backward()).

All these combinations give rise to over 162, 000 tests (note also that the exact number of tests varys by architecture due to the differing vector unit lengths). This large number of tests ensures all the separately optimised code paths for the various combinations of run-time options are covered. We note that the tests take quite some time to run (e.g. potentially 30-60 minutes on a laptop), precisely because the gold master R code is run to provide the ground truth for these tests.

If a user wishes to confirm correctness on their particular platform, they can be run with the following commands:
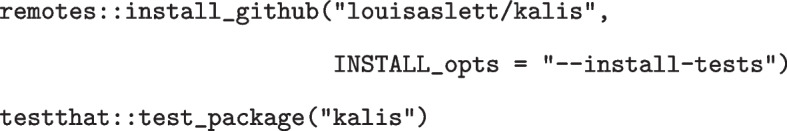


## Results

We provide a brief overview of some example performance figures, though due to the highly tuned nature of kalis, the exact performance you can expect will be heavily dependent on your exact computer architecture and resources.

**Fig. 2:**
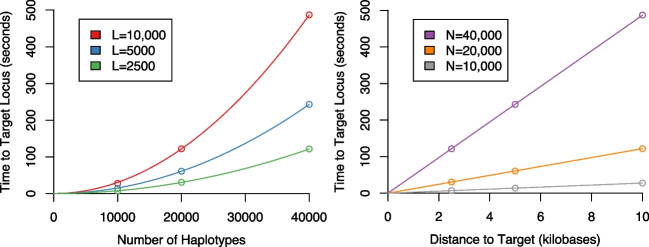
kalis shows the expected order $N^2$ and order *L* scaling of the LS model. Computed on an Amazon Web Services c4.8xlarge instance (36 vCPUs, 60 GB of RAM)

First, it is important to note we do *not* claim to have altered the scaling properties of the LS model, only that we provide an implementation which is highly optimised within the scaling constraints inherent to the model. As such, Fig. 2 demonstrates that kalis indeed inherits the ${\mathcal {O}}(N^2)$ and ${\mathcal {O}}(L)$ properties of the original LS model.

We turn now to the benefits kalis does provide.

**Fig. 3:**
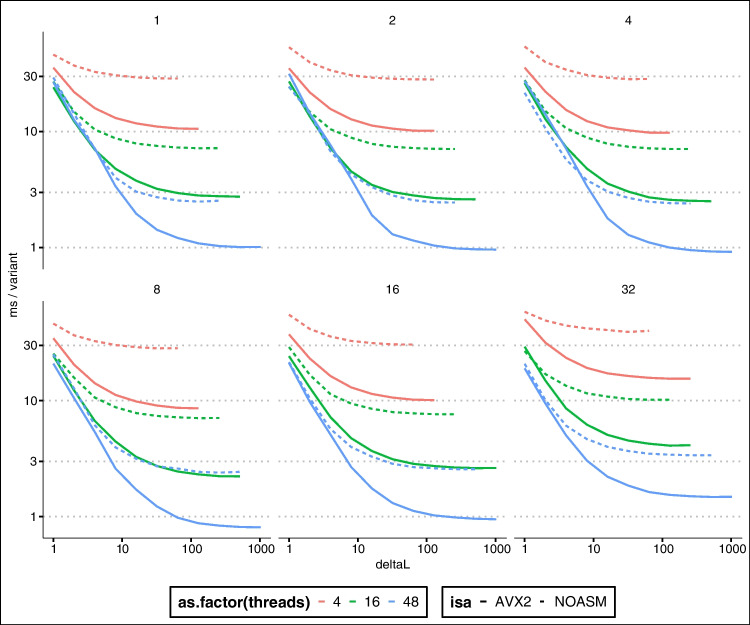
Log-log plot of milliseconds per variant performance (*y*-axis) of the forward algorithm on 10,000 haplotypes, against the number of variants propagated (*x*-axis). Each panel is a different loop unrolling depth (panel title gives loop unrolling level). Line colour denotes number of CPU threads, whilst a dashed line indicates vanilla C and a solid line indicates hand-coded AVX2 instructions. In total, using AVX2, 48 threads, and loop unrolls to depth 8, it takes less than 10 seconds to propagate a $10000 \times 10000$ forward table over 10,000 variants

Firstly, for some of the reasons highlighted in the previous Section, kalis exhibits accelerated performance when propagating the forward/backward recursions over more extended stretches of the genome. This is because every effort has been made to be cache efficient, so that when more than a single variant step is taken, the strong cache locality design ensures that we are not memory bandwidth limited. This effect can be seen quite dramatically in Fig. 3 by the rapid decrease in compute time per variant as longer stretches are propagated.

Secondly, the hard-coded loop unrolling functionality which can be controlled at compile time by the user can be seen to be beneficial in Fig. 3. Clearly excessive loop unrolling is harmful, with depth 32 unrolls actually being substantially slower than no unrolling. However, unrolling to depth 8 does give a clear improvement. The best choice of unrolls will be both problem and architecture dependent, so we recommend testing different unroll levels on the target problem before performing long compute runs.

Figure 3 also illustrates that the hand-designed use of low-level vector SIMD instructions is not superfluous, with substantial speed-up afforded by their use (the difference between dashed and solid lines of the same colour).

**Fig. 4:**
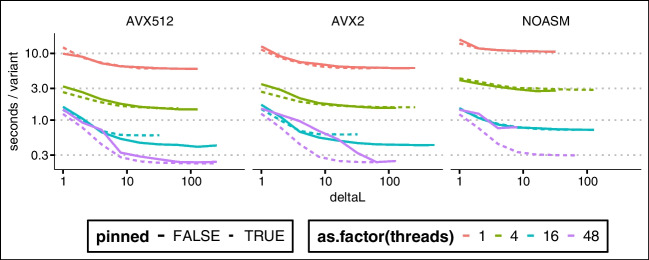
Log-log plot of seconds per variant performance (*y*-axis) of the forward algorithm on 100,000 haplotypes, against the number of variants propagated (*x*-axis). Each panel is a different instruction set (AVX-512/AVX2/none). Line colour denotes number of CPU threads, whilst a dashed line indicates pinned threads and a solid line indicates no thread pinning. In total, using AVX-512, 48 threads, and pinned threads, it takes less approximately 38 minutes to propagate a $100000 \times 100000$ forward table over 10,000 variants

Finally, Fig. 4 shows that in certain very large problem settings kalis’ ability to pin threads can make a substantial difference. In this setting, AVX2 showed the greatest benefit from eliminating context switching, ensuring that the cache is not invalidated by threads migrating between cores. The lack of substantial difference between AVX2 and AVX-512 here once thread pinning is employed calls for some investigation, though this may be a result of thermal/power throttling which is known to occur especially for AVX-512 heavy code [17].

These performance examples again highlight the importance of pilot benchmark runs with different configurations of instruction set and unroll settings before embarking on long compute runs to ensure the greatest compute efficiency is achieved for a given problem and compute architecture.

### Benchmarking comparison

We performed two benchmarking experiments to compare the implementations of the forward and backward algorithms in kalis to those in Relate [2]. While several other leading software suites, including BEAGLE [4] and IMPUTE [5], use high performance implementations of the LS model, we chose to compare to Relate because it is explicitly optimized to target locus-specific $N \times N$ genetic distance matrices analogous to those produced by kalis. We based all of our benchmarks on the same set of haplotypes, taken from the 1000 Genomes Project [18], as used in our real-data example which follows below. The data include 5008 haplotypes observed at 29193 variants.

kalis can perform the forward and backward recursions under either the original LS model (the default) or the derived allele haplotype copying model if use.speidel=TRUE is passed to the Parameters() function. Since Relate only computes these recursions for the derived allele copying model, it can exploit the asymmetry in the emission kernel based on the derived allele orientation of each variant. When painting a given recipient haplotype as a mosaic of donor haplotypes, this allows Relate to effectively skip all variants where a recipient haplotype does not carry the derived allele. This acceleration cannot be applied to the original LS model, which kalis was primarily designed for, because the symmetric emission kernel requires both the forward and backward algorithms to iterate over every variant for every recipient haplotype. Even with the derived allele copying model activated, kalis will still visit every variant for every recipient haplotype.

Accordingly, we found that the forward and backward recursions were approximately $4\times$ faster using Relate rather than using kalis. However, if Relate visits every locus, as would be necessary to compute the original LS model, we found that the forward and backward recursions were approximately $6\times$ faster using kalis rather than using Relate. This demonstrates the benefit of the low-level optimisations made in kalis. In principle kalis could also employ the same optimisation as Relate and visit only derived sites for every recipient haplotype. We consider this an exciting avenue of future research. Otherwise, kalis and Relate would be expected to share similar algorithmic scaling properties in data size.

Full details of how this benchmarking was performed are provided in Additional file 1, Section D.

### Real-data example: recent selection for lactase persistence

*LCT* is a gene on chromosome 2 that encodes lactase, the enzyme responsible for the breakdown and digestion of lactose, the sugar commonly found in milk. Ancestral humans had a regulatory ‘switch’ on chromosome 2 that stops lactase production after infancy when children would be weaned off breast milk. Mutations that disrupt this switch allow lactase production to persist into adulthood, conferring a lifelong ability to extract energy from milk [19]. Such mutations have arisen independently at least twice in human history, in Europe and in East Africa, and are among the strongest examples of recent positive natural selection in humans [20, 21]. These mutations have been shown to spread across standard human population boundaries. For example, [22] used another implementation of the LS model to compare haplotypes at the *LCT* locus sampled from the West African Fula population to haplotypes collected from across Europe and Asia as part of the 1000 Genomes project [18]. They found that the genetic distance between Fulani haplotypes and Eurasian haplotypes was unusually small at the *LCT* locus. With some further analysis, they interpreted this as evidence that a European haplotype conferring lactase persistence became prevalent within the West African Fula population due to recent natural selection sometime over the past two thousand years.

**Fig. 5:**
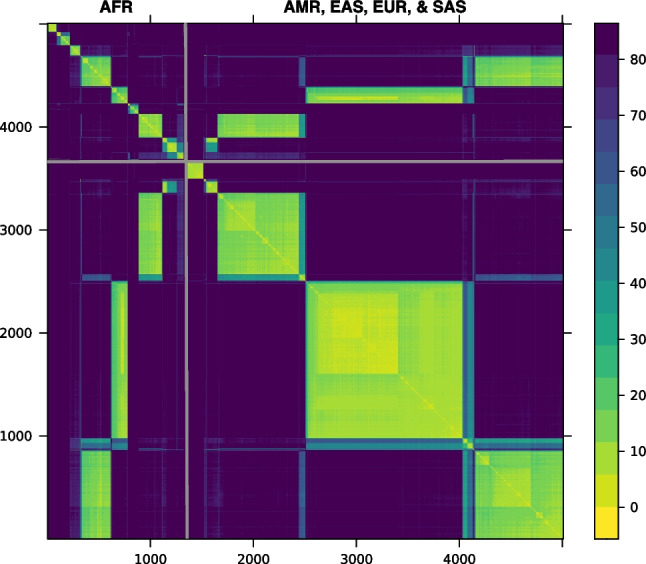
Distance matrix among 5008 haplotypes calculated at rs4988235, upstream of *LCT*. African haplotypes are clustered in the upper left corner and separated by grey lines from non-African haplotypes from the Americas (AMR), East Asia (EAS), Europe (EUR), and SAS (South Asia). The scale on the right maps the colours to distances

Although it is difficult to directly replicate [22] since the Fulani samples they studied are not a part of the 1000 Genomes project, we take inspiration from their analysis. Here we present a small example using kalis to informally investigate whether there is evidence of recent gene-flow from Eurasia into any of the African populations in the 1000 Genomes dataset at the lactase locus. We run kalis on 5008 haplotypes from the 1000 Genomes Phase 3 release to revisit the haplotype structure around *LCT*; the haplotypes are sampled from 26 sub-populations all over the world [18]. Figure 5 shows a clustered version of a distance matrix, calculated as in Eq. (4), at a variant in the regulatory region of *LCT* (rs4988235). To see if we could observe a pattern of gene-flow into or out of Africa similar to what was observed by [22], we use average pairwise linkage [23] to cluster the African haplotypes separately from the non-African haplotypes. In Fig. 5, distances between African haplotypes are shown in the upper left corner; non-African haplotypes, in the lower right corner.

Rather than 26 clusters reflecting the 26 sampled human populations, we see that there are three very distinct lactase haplotypes that are common both within and outside Africa. This suggests that these three haplotypes, under strong positive selection pressure, recently spread across population boundaries and presumably confer lactase persistence. We cannot confirm whether any of these three haplotypes correspond to the one identified in the Fulani by [22]. These three haplotypes are not the only structure we see: in the upper left corner of the African (AFR) block we see some haplotypes that are only found inside Africa; and in the non-African block, a haplotype that is only found outside Africa. We can also see some sub-structure within the clear haplotype blocks.

The code to reproduce this example is available in the examples directory of repository associated with this paper (https://github.com/louisaslett/kalis-bmc), as a vignette in the package (if vignettes built at install time), or directly at the kalis package website https://kalis.louisaslett.com/articles/lct_example.html

## Discussion

In Additional file 2, we introduce the package from a user perspective, from package installation right through to decoding a single variant position in R using kalis.

There are many avenues for future research in developing kalis. On the model side, for example, allowing for different recombination rates between sub-populations as done in fastPHASE [24] would be a natural extension.

On the computational side, ARM scalable vector extensions [25] represent an interesting new approach to SIMD instruction sets, where the width of instructions need not be hard coded prior to compilation. At present it is not widely available, but as this rolls out, it would be natural to extend kalis to enable targeting this new instruction set.

An important utility extension is expanding the file formats that kalis can natively read via CacheHaplotypes(), to enable simpler and more streamlined software pipelines when bioinformaticians incorporate kalis into their workflows.

Additionally, during development of kalis we have been congnisant of the potential interest in using the core C code from other languages. Therefore all core computational C code has been kept as low-dependency as possible, and in particular has no dependencies on R or any other external libraries. We hope in future to release a pure C library, or to provide other language bindings directly.

Finally, a future avenue of potential development is extension of kalis to support GPU or tensor cards. Note that it was an explicit design choice to initially target CPU SIMD extensions, since the vast majority of University high performance computing clusters have a huge amount of untapped compute power in this form, but often much more limited availability of specialist extension cards. Therefore, by pushing performance as extensively as possible via CPU only means, we provide the greatest potential impact for end users. This does not preclude future versions adding support for add-on compute cards.

## Conclusion

kalis provides a R interface to a highly optimized C implementation of the LS model that enables local ancestry, selection, and associations studies in modern large genomic datasets.

## Availability and requirements

Project name: kalis
Project home page: https://kalis.louisaslett.com/
Operating system(s): Linux, MacOS, Windows
Programming language: R, C
Other requirements: R ($$\ge$$ 3.5.0)
License: GPL ($$\ge$$ 3)
Any restrictions to use by non-academics: None beyond GPL ($\ge$ 3).

### Supplementary Information

**Additional file 1.** An appendix containing full derivations of mathematical reformulation; software installation details; HDF5 file format specifications; and additional benchmarking details.

**Additional file 2.** A user guide to the kalis R package.

## Data Availability

The package source code repository is at https://github.com/louisaslett/kalis. All scripts for reproducing the results of this paper are available in this repository https://github.com/louisaslett/kalis-bmc. The two external dependencies are: 1000 Genomes data which are available for download from https://www.internationalgenome.org/; and the msprime simulator, which may be downloaded from https://tskit.dev/software/msprime.html.

## Abbreviations

LS model: Li & Stephens model
HMM: hidden Markov model
SIMD: single instruction, multiple data

## Footnotes

1 Certain modern CPUs do not require specific alignment to be able to load memory to SSE/AVX registers, but for maximum compatibility we honor the alignment anyway.

2 Measured under R 4.2.2
