# Supplementary material for: kalis: a modern implementation of the Li & Stephens model for local ancestry inference in R
Source: BMC Bioinformatics. 2024 Feb 28;25:86. doi: 10.1186/s12859-024-05688-8 (PMC10900616; doi:10.1186/s12859-024-05688-8)
Supplement: Supplementary file 1 — Additional file 1. An appendix containing full derivations of mathematical reformulation; software installation details; HDF5 file format specifications; and additional benchmarking details. [file 12859_2024_5688_MOESM1_ESM.pdf]

kalis: A Modern Implementation of the Li & Stephens Model for Local Ancestry Inference in R
Appendix

Louis J.M. Aslett & Ryan R. Christ

A Mathematical details of HMM reformulation

A.1 Rearrangement of the forward recursion

Starting with (7) from the main paper, we have

$$\begin{aligned}
\tilde{\alpha}_{\cdot i}^{\ell} &\leftarrow \theta_{\cdot i}^{\ell}\left(\left(1-r^{\ell-1}\right)\tilde{\alpha}_{\cdot i}^{\ell-1}+r^{\ell-1}F_i^{\ell-1}\Pi_{\cdot i}\right)\\
\frac{\tilde{\alpha}_{\cdot i}^{\ell}}{F_i^{\ell-1}} &\leftarrow \theta_{\cdot i}^{\ell}\left(\left(1-r^{\ell-1}\right)\frac{1}{F_i^{\ell-1}}\tilde{\alpha}_{\cdot i}^{\ell-1}+r^{\ell-1}\Pi_{\cdot i}\right)\\
\frac{\tilde{\alpha}_{\cdot i}^{\ell}}{F_i^{\ell-1}} &\leftarrow \theta_{\cdot i}^{\ell}\left(\left(1-r^{\ell-1}\right)\frac{F_i^{\ell-2}}{F_i^{\ell-1}}\frac{\tilde{\alpha}_{\cdot i}^{\ell-1}}{F_i^{\ell-2}}+r^{\ell-1}\Pi_{\cdot i}\right)\\
\alpha_{\cdot i}^{\ell} &\leftarrow \theta_{\cdot i}^{\ell}\left(\left(1-r^{\ell-1}\right)\frac{F_i^{\ell-2}}{F_i^{\ell-1}}\alpha_{\cdot i}^{\ell-1}+r^{\ell-1}\Pi_{\cdot i}\right)
\end{aligned}$$

Since $\frac{F_i^{\ell-2}}{F_i^{\ell-1}}=\left(\frac{\sum_j\tilde{\alpha}_{ji}^{\ell-1}}{F_i^{\ell-2}}\right)^{-1}=\left(\sum_j\alpha_{ji}^{\ell-1}\right)^{-1}$, we arrive at (10).

A.2 Rearrangement of the backward recursion

Starting with (8) we have

$$\begin{aligned}
\tilde{\beta}_{\cdot i}^{\ell} &\leftarrow \left(1-r^{\ell}\right)\tilde{\beta}_{i\cdot}^{\ell+1}\theta_{\cdot i}^{\ell+1}+r^{\ell}G^{\ell}\\
\frac{\tilde{\beta}_{\cdot i}^{\ell}}{G^{\ell}} &\leftarrow \left(1-r^{\ell}\right)\frac{1}{G^{\ell}}\tilde{\beta}_{\cdot i}^{\ell+1}\theta_{\cdot i}^{\ell+1}+r^{\ell}\\
\frac{\tilde{\beta}_{\cdot i}^{\ell}}{G^{\ell}} &\leftarrow \left(1-r^{\ell}\right)\frac{G^{\ell+1}}{G^{\ell}}\frac{\tilde{\beta}_{\cdot i}^{\ell+1}}{G^{\ell+1}}\theta_{\cdot i}^{\ell+1}+r^{\ell}\\
\beta_{\cdot i}^{\ell} &\leftarrow \left(1-r^{\ell}\right)\frac{G^{\ell+1}}{G^{\ell}}\beta_{\cdot i}^{\ell+1}\theta_{\cdot i}^{\ell+1}+r^{\ell}
\end{aligned}$$

Since $\frac{G^{\ell+1}}{G^{\ell}}=\left(\frac{\sum_j\tilde{\beta}_{ji}^{\ell+1}\theta_{ji}^{\ell+1}\Pi_{ji}}{G_i^{\ell+1}}\right)^{-1}=\left(\sum_j\beta_{ji}^{\ell+1}\theta_{ji}^{\ell+1}\Pi_{ji}\right)^{-1}$, we arrive at (12).

A.3 Numerical considerations: avoiding `NaN`

The forward and backward recursions as written in (10) and (12) are susceptible to three main categories of numerical instability: element underflow, total underflow, and overflow. By element underflow, we refer to the situation where a subset of elements in $\alpha^{\ell}_{\cdot i}$ or $\beta^{\ell}_{\cdot i}$ underflow to zero for a given ℓ. This becomes more likely to occur if there are zero (or near zero) entries in ρ, μ, or Π (excepting the diagonal, which must be zero). Element underflow effectively reinitializes the recursion for the donor haplotypes corresponding to the elements where the underflow occurs, causing the HMM to lose track of how similar those donor haplotypes are to the recipient haplotype leading up to the variant where the underflow occurs. While element underflow results in a loss of information about the relative likelihood of the recipient copying from genetically distant donors, the relative likelihood of copying similar donors is retained. Element underflow will not cause either recursion to fail.

However, underflow can cause catastrophic failure if it causes either:

$$\sum_j \alpha^{\ell-1}_{ji} \qquad \text{or} \qquad \sum_j \beta^{\ell+1}_{ji}\theta^{\ell+1}_{ji}\Pi_{ji}$$

to evaluate to zero at a given ℓ, which we refer to as total underflow. In these cases, entire columns of `fwd$alpha` or `bck$beta` will be `NaN` (except for the diagonal which is always 0). While this means that the user cannot continue the recursion with the current set of haplotypes and parameters, total underflow is easy to catch since the `NaN`s will usually break downstream pipelines.

Internally, **kalis** takes several measures to help reduce the risk of total underflow. For example, **kalis** calculates $\theta^{\ell}_{\cdot i}$ as

$$\theta^{\ell}_{\cdot i} = \left(1 - H^{\ell}_{\cdot i}\right)(1 - 2\mu) + \mu.$$

Note, that even for μ well below machine precision, this sets θ^{ℓ}_{ji} to μ for haplotypes j, i that mismatch at ℓ rather than setting θ^{ℓ}_{ji} to 0. This in turn helps prevent $\theta^{\ell}_{\cdot i}$ from being set to zero, resulting in total underflow. Despite the precautions taken in **kalis**, we recommend that users remove private mutations that appear on only one haplotype (singletons) before loading haplotypes into the **kalis** cache. These private mutations tend not to be informative about the relationships between haplotypes and removing them can help prevent total underflow, especially at variants with small μ.

In addition to removing singletons, users may consider avoiding parameter choices that place many zero (or near zero) entries in ρ, μ, or Π to help prevent or remedy total underflow. Setting some entries of ρ zero, for a non-recombining segment of genome, or μ to zero, for very important variants that all potential donors must share with a recipient, is often biologically meaningful and should be safe in most applications. However, setting entries of Π to zero requires slightly more caution because this can easily cause total underflow if the prior copying probabilities strongly conflict with the observed haplotype similarity over a genomic interval (prior-likelihood mismatch). Furthermore, zero (or very near zero) prior copying probabilities can cause $\sum_j \beta^{\ell+1}_{ji}\theta^{\ell+1}_{ji}\Pi_{ji}$ to evaluate to `Inf` which we refer to as total overflow when there is prior-likelihood mismatch. Like total underflow, total overflow is easily detectable since it leads to `NaN`s on the next backward iteration. The forward recursion is not susceptible to total overflow because every α^{ℓ}_{ji} is less than or equal to 2[†]. Using prior copying probabilities that reflect the

[†]In a different implementation, one may be tempted to divide both sides of the forward recursion and backward recursion by ρ. This would help prevent underflow by allowing α to make full use of the range of double precision numbers and have the added benefit of slightly increased performance (by eliminating a multiply AVX instruction). However, rescaling by ρ makes it impossible to tackle problems where ρ^{ℓ} is zero for some ℓ. Even if we restrict

observed haplotypic similarity or simply resorting to uniform prior copying probabilities for Π (the default) should be safe in most applications.

B Installation help

There are two key features that can only be set at package compile time: the SIMD instruction set to target, and how deeply to unroll the innermost loop in the **kalis** core. This appendix explains how to set these options at compile time.

B.1 Manually controlling instruction set

When compiling **kalis** it will by default attempt to auto-detect the best available SIMD instruction set to use. However, if this auto-detection fails, or if you wish to force the use of an inferior (or no) SIMD instructions then you can use a compiler flag to manually direct **kalis**.

The supported flags are:

Flag	Instruction sets used
`NOASM`	Forces pure C, no special instruction set intrinsics used
`AVX2`	Enables intrinsics: AVX2, AVX, SSE4.1, SSE2, FMA and BMI2
`AVX512`	Enables intrinsics: AVX-512, AVX2, SSE2 and BMI2
`NEON`	Enables intrinsics: ARM NEON and NEON FMA

They are used by setting the configure variable `FLAG=1`, where `FLAG` is one of the options in the above table. For example, to force the use of no special instruction set whilst still compiling against the native architecture, one would compile with:

```
remotes::install_github("louisaslett/kalis", configure.vars =
        c(kalis = "PKG_CFLAGS='-march=native -mtune=native -O3' NOASM=1"))
```

Note that the most restrictive instruction set is all that need be defined: that is, the availability of AVX2 implies the availability of the other listed instruction sets, and likewise for AVX-512 and NEON. Hence, there is no need to verify the availability of other instruction sets.

On a Mac, you can check if you have any of these instruction sets by running the following at a Terminal, the lines ending `1` indicating support (missing lines or those ending `0` indicate no support):

```
sysctl -a | egrep "^hw.optional.(avx2|avx5|neon)+"
```

On most flavours of Linux you can check if you have AVX2 or AVX-512 instruction sets by running (no output indicates no support, otherwise look for the highlighted `avx` text):

```
egrep --color -i "avx[^ ]*" /proc/cpuinfo
```

On most flavours of Linux you can check if you have NEON instruction set by running (no output indicates no support):

```
egrep --color -i "(neon|asimd[^ ]*)" /proc/cpuinfo
```

$\rho^\ell > 0$, the rescaling makes the forward recursion susceptible to total overflow since it raises the upper bound on α from 2 to $1 + \left(\min_\ell \rho^\ell\right)^{-1}$. Similarly, it makes the backward recursion more susceptible to overflow by raising the upper bound on β from $1 + \left(\min_{j,i} \Pi_{ji}\right)^{-1}$ to $1 + \left(\min_{\ell,j,i} \rho^\ell \Pi_{ji}\right)^{-1}$.

B.2 Core loop unrolling

Loop unrolling can improve performance for critical deeply nested loops. This occurs either because for a sufficiently optimised loop, the loop increment count comprises a substantial proportion of the computation of each iteration, or because by unrolling the compiler (and CPU at run time) can reason about between iteration dependency better leading to better instruction ordering and potential instruction level parallelism.

By default **kalis** will unroll loops to depth 4, which we have tested to be a reasonable default for many machines and problem sizes. However, the optimal value will vary both by the particulars of a CPU/memory architecture and by problem size (in N). Therefore, if **kalis** represents a performance critical section of your workflow, we recommend running real benchmarks for a variety of unroll depths on a problem of your target size on the machine you will deploy to.

In order to set the unroll depth, you need to pass the `UNROLL` configure variable. Note that only powers of 2 are supported.

For example, to double the default unroll depth to 8, use the following at compile time:

```
remotes::install_github("louisaslett/kalis", configure.vars =
        c(kalis = "PKG_CFLAGS='-march=native -mtune=native -O3' UNROLL=8"))
```

Note that the unroll flag and the target SIMD instruction set flags of Appendix B.1 can be set together with a space separator between them.

C HDF5 file format

HDF5 [4] is a format designed to handle large quantities of data in an efficient manner. **kalis** supports loading data from HDF5 in the format specified here, with the option to depend on either CRAN package **hdf5r** [3] or Bioconductor [2] package **rhdf5** [1].

For HDF5 files, **kalis** expects a 2-dimensional object named `/haps` at the root level of the HDF5 file. Haplotypes should be stored in the slowest changing dimension as defined in the HDF5 specification (**note:** different languages treat this as rows or columns: it is 'row-wise' in the C standard specification, or 'column-wise' in the **rhdf5** specification). If the haplotypes are stored in the other dimension then simply supply the argument `transpose = TRUE` when calling `CacheHaplotypes()`, although this may incur a small penalty in the time it takes to load into cache. If you are unsure of the convention of the language in which you created the HDF5 file, then the simplest approach is to simply load the data with `CacheHaplotypes()` specifying only the HDF5 file name and then confirm that the number of haplotypes and their length have not been exchanged in the diagnostic output which **kalis** prints.

The format also allows named IDs for both haplotypes and variants in the 1-dimensional objects `/hap.ids` and `/loci.ids`, also in the root level of the HDF5 file.

C.1 Working with this format in R

Since the format described above is not standard, **kalis** provides two utility functions for working with it. `WriteHaplotypes()` enables using R to create the requisite format from a standard matrix, and `ReadHaplotypes()` allows reading into R (rather than the internal **kalis** cache) from this format.

Assume that you have imported your haplotype data into the R variable `myhaps`, with variants in rows and haplotypes in columns (so that `myhaps` is an $L \times N$ matrix consisting of only `0` or `1` entries). Then to write this out to the HDF5 file `~/myhaps.h5`,

```
R> WriteHaplotypes("~/myhaps.h5", myhaps)
```

You may additionally wish to store the names of haplotypes or variants alongside the haplotype matrix for self documentation purposes. Assume you have defined `hapnm`, a character vector of length N, and `varnm`, a character vector of length L, then you may instead call,

```
R> WriteHaplotypes("~/myhaps.h5", myhaps,
                   hap.ids = hapnm, loci.ids = varnm)
```

You can verify the content by reading this file back into R directly as:

```
R> myhaps_file <- ReadHaplotypes("~/myhaps.h5")
```

You should find `all(myhaps == myhaps_file)` is `TRUE`. For large problems, you may want to read just one named variant. For example, imagine you had saved the variant names using the second `WriteHaplotypes()` function call above, and that one of those variant names was `"rs234"` (genetic variant associated with lactase persistence), then you can read all haplotypes for this variant with,

```
R> lactase_haps <- ReadHaplotypes("~/myhaps.h5",
                                  loci.ids = "rs234")
```

Once you are happy the HDF5 file is setup correctly, you can restart your R session to ensure all memory is cleared and then load your haplotype data directly to the optimised **kalis** cache by passing the file name,

```
R> CacheHaplotypes("~/myhaps.h5")
```

D Benchmark comparison details

We consider two benchmarking experiments to compare the implementation of the forward and backward algorithms in **kalis** to those in Relate.

As mentioned in the main paper, Relate is designed to target the derived allele haplotype copying model where there is asymmetry in the emission kernel based on the derived allele orientation of each variant. When painting a given recipient haplotype as a mosaic of donor haplotypes, this allows Relate to effectively skip all variants where a recipient haplotype does not carry the derived allele. By propagating over different subsets of variants in this way, the Relate paper reported an order-of-magnitude speed up (*"In practice this can make the application of the algorithm* > 10 *times faster even for modest samples of a few thousand individuals,"* end §3.4 of supplementary note in [2]). However, this approach cannot be applied to the original LS model because the emission kernel's symmetry effectively requires every variant to be accounted for when painting each recipient haplotype. While **kalis** was optimized for the original LS model, the derived allele copying model is available using the flag `use.speidel = TRUE` (see the `Parameters()` function). Note that even with the derived allele copying model activated, **kalis** will still visit every variant for every recipient haplotype; in principle **kalis** could also employ the same optimisation as Relate and visit only derived sites for every recipient haplotype. We consider this an exciting avenue of future research.

By default Relate visits an internally determined schedule of sites at which to output forward and backward probabilities, to eliminate the IO time required we modified one of the binary parameter files produced by Relate to run painting without any outputting any intermediate sites.

Hence, we designed two benchmarking experiments. The first comparing speed while targeting the derived allele copying model; the second, the original LS model. Since the Relate license bars modification of the source code, we could not simply inject timing code around the forward and backward recursions.

For the purposes of benchmarking we created two synthetic datasets from the 1000 Genomes data used in our lactase example (5008 haplotypes observed at 29193 variants), `lct0` and `lct1`. To generate `lct0`, we replaced the last haplotype in the dataset with all 0s except for a single 1 at both ends. We then generated `lct1` by again modifying the last haplotype to be all 1s except for a single 0 at the middle variant (needed to overcome a restriction in Relate whereby not all variants may be 1 for a single haplotype). This facilitated benchmarking because in `lct1` all of the 1s in the last haplotype forces Relate to visit all variants (except for the middle variant) when painting the last haplotype. Hence, taking the difference between the run time for Relate on `lct0` versus `lct1` provides an estimate of the amount of time required for Relate's forward and backward algorithm to iterate over all variants for a single haplotype.

In both cases we used the same recombination map, with very high spikes inserted between the first and second variant as well as between the last and penultimate variant. These spikes forced the forward and backward copying probabilities obtained at each end of the haplotypes to be identical when running Relate on `lct0` and `lct1`. This is important because Relate uses an on-the-fly compression method when writing the forward and backward probabilities to disk, and this recombination map ensures the same data is compressed in both runs eliminating a potential source of bias. Finally, we also overrode the default file writing schedule so that only probabilities at either end of the haplotypes were outputted to minimise I/O.

First, the `lct0` run in Relate was performed 10 times, with mean run time 234.4937 secs, 95% CI $(234.4141, 234.5733)$. Performing the analagous experiment for `lct0` in **kalis** corresponds to propagating the forward and backward algorithms over the entire chromosome for all N haplotypes. In **kalis** this yielded a mean run time 20.5058 secs, 95% CI $(20.5009, 20.5108)$. It should be noted that **kalis** automatically took advantage of the 48 core CPU, whilst Relate does not do multi-threading of forward or backward pass. With the roughly linear scaling, this means if forced to run single core **kalis** would be about $4\times$ slower, the difference arising because Relate skips many variants by default.

We next explore what advantage the software engineering in **kalis** provides when targetting the original LS model under which all sites must be visited. To do so we examine the difference between `lct0` and `lct1` runs under Relate and compare it to **kalis** painting a single haplotype (the last one) for `lct1`. The experiments were run 10 times resulting in a mean time of 234.4937 secs for `lct0` and 235.2122 secs for `lct1`, with unpaired 95% confidence interval for the difference of $(0.6336, 0.8034)$ with mean 0.7185 secs. This represents our estimate of the amount of time required for Relate's forward and backward algorithm to iterate over all variants for a single haplotype (specifically, the last haplotype in `lct1`). This compares to **kalis** taking a mean run time of 0.1236 secs with 95% confidence interval $(0.1216, 0.1257)$. This corresponds to an average $5.8\times$ improvement under **kalis**.

Thus, in the Relate-native setting Relate performs better than **kalis**, while in the **kalis**-native setting **kalis** performs better than Relate.

References

[1] Fischer, B., Smith, M., and Pau, G. *rhdf5: R Interface to HDF5*, 2022. R package version 2.42.0.

[2] Gentleman, R. C., Carey, V. J., Bates, D. M., Bolstad, B., Dettling, M., Dudoit, S., Ellis, B., Gautier, L., Ge, Y., Gentry, J., Hornik, K., Hothorn, T., Huber, W., Iacus, S., Irizarry, R., Leisch, F., Li, C., Maechler, M., Rossini, A. J., Sawitzki, G., Smith, C., Smyth, G., Tierney, L., Yang, J. Y., and Zhang, J. Bioconductor: open software development for computational biology and bioinformatics. *Genome Biology 5*, 10 (2004), R80.

[3] Hoefling, H., and Annau, M. *hdf5r: Interface to the 'HDF5' Binary Data Format*, 2022. R package version 1.3.7.

[4] The HDF Group. Hierarchical Data Format, version 5, 1997-2022.
