# Supplementary material for: kalis: a modern implementation of the Li & Stephens model for local ancestry inference in R
Source: BMC Bioinformatics. 2024 Feb 28;25:86. doi: 10.1186/s12859-024-05688-8 (PMC10900616; doi:10.1186/s12859-024-05688-8)
Supplement: Supplementary file 2 — Additional file 2. A user guide to the kalis R package. [file 12859_2024_5688_MOESM2_ESM.pdf]

kalis: A Modern Implementation of the Li & Stephens Model for Local Ancestry Inference in R
User Guide

Louis J.M. Aslett & Ryan R. Christ

Here we introduce the package from a user perspective, from package installation right through to decoding a single variant position. **kalis** is an R package [8], with all time critical code developed in C [4] with extensive use of low-level SIMD instructions. These full technical details are presented in the main text for the interested reader, but from a user perspective these high performance implementation details are hidden behind a user-friendly API detailed below.

Installing kalis

kalis may be made available on CRAN in future, but users on MacOS and Windows would need to be aware that out of necessity any CRAN binary would be compiled for maximum compatibility, not maximum performance. We *strongly* recommend compiling the package from source code if you plan to work with large haplotype sets (eg $N > 1000$).

Compiling from source for maximum performance

To install directly from Github, compiling from source, it is easiest to use the **remotes** package [1]. *This is the recommended installation method since bug fixes are pushed immediately to Github.*

```
remotes::install_github("louisaslett/kalis", configure.vars =
        c(kalis = "PKG_CFLAGS='-march=native -mtune=native -O3'"))
```

If the above installation command works correctly, the `PKG_CFLAGS` setting will be reported back to you. In most cases, **kalis** will then be able to auto-detect the vector instruction set of your CPU and this will be reported too. For example, you would see the following near the start of the console output on a modern Intel CPU which supports AVX2:

```
Using PKG_CFLAGS=-march=native -mtune=native -O3
Using PKG_LIBS=-lz
AVX2 family of instruction set extensions will by used (auto-detected).
```

If you have an error in passing the `PKG_CFLAGS` setting, the first line will instead read:

```
Using PKG_CFLAGS=
```

If auto-detection of the instruction set has failed (or if the `PKG_CFLAGS` was not passed properly), then the third line will instead read:

```
No special assembly instruction set extensions will by used (auto-detected).
```

It is possible to override the auto-detection and manually direct **kalis** which instruction set to use, please see the Appendix (Additional file 1) for details.

Finally, when you load the **kalis** package, you will also receive a diagnostic message confirming the status of the compiled code. For example, the aforementioned Intel CPU compilation provides the following diagnostic on loading:

```
R> library("kalis")

Running in 64-bit mode using x86-64 architecture.
Loops unrolled to depth 4.
Currently using AVX2, AVX, SSE4.1, SSE2, FMA and BMI2 CPU instruction set
  extensions.
```

Advanced users should note that **kalis** will respect `CFLAGS` settings in `~/.R/Makevars`, so be alert to any compiler flags set there that may conflict with the above installation commands. Advanced users may also be interested to benchmark performance under different levels of loop unrolling: manually controlling this setting at compile time is described in the Appendix (Additional file 1).

Package overview

In the *v*1 release of **kalis** there are 18 functions which enable computation of the LS model. These are briefly summarised in Table 1, grouped by the task under which the function falls.

**Function**	**Purpose**
Load and Inspect Haplotypes	
`CacheHaplotypes`	Reads haplotype data into internal **kalis** format
`CacheSummary`	Prints current state of the haplotype cache
`ClearHaplotypeCache`	Frees internal cache memory
`N` and `L`	Retrieve number of phased haplotypes, N, and sites, L
`QueryCache`	Retrieve haplotypes from the internal memory cache
Initialise HMM	
`MakeBackwardTable`	Constructs $N \times N$ backward matrix for N HMMs
`MakeForwardTable`	Constructs $N \times N$ forward matrix for N HMMs
`Parameters`	Define LS model parameters ρ, μ, Π and compute options
HMM Propagation	
`Backward`	Executes the backward recursion of Equations (11) and (12)
`Forward`	Executes the forward recursion of Equations (9) and (10)
Decode HMM	
`DistMat`	Computes distances per Equation (4)
`PostProbs`	Compute posterior marginal probabilities, Equation (13)
Utilities	
`CalcRho`	Compute recombination probabilities, ρ, Equation (3)
`CopyTable`	Creates a fully cloned (deep) copy of table
`ReadHaplotypes`	Load haplotypes from HDF5 format into an R object

`ResetTable`	Efficiently wipe a forward/backward table for reuse
`WriteHaplotypes`	Save haplotypes from binary R matrix into HDF5 format

Table 1: Exported **kalis** functions grouped by task. Equation numbers reference the main paper. See corresponding detailed help files in the package for argument and return value information.

Full details of these functions can be found in the package documentation, or at the package website, `https://kalis.louisaslett.com/`.

In the remainder of this section we provide detailed comments on the steps involved in using **kalis** to compute the posterior marginal probabilities (Equation (13)) or distances (Equation (4)), with numerous pertinent asides to aid using the package efficiently. These steps are broken down as: (i) loading the haplotype data from R or disk; (ii) setting the model parameters ρ, μ, Π; (iii) initialising the N HMMs; (iv) running the forward/backward algorithms; (v) computing the posterior marginal probabilities and distances.

Loading haplotype data

In order to demonstrate how to use **kalis**, the package comes with a toy data set of 300 simulated haplotypes, `SmallHaps`.

```
R> library("kalis")

Running in 64-bit mode using x86-64 architecture.
Loops unrolled to depth 4.
Currently using AVX2, AVX, SSE4.1, SSE2, FMA and BMI2 CPU instruction set
  extensions.

R> data("SmallHaps")
```

This simulated dataset is stored as an $L = 400$ by $N = 300$ matrix with binary entries, as can be seen by inspecting with `str()`,

```
R> str(SmallHaps)

int [1:400, 1:300] 0 0 0 0 0 0 0 1 0 0 ...
```

In order to run the LS model, haplotype data must first be loaded into the internal optimised cache using the `CacheHaplotypes()` function. This function accepts an integer R matrix with $\{0, 1\}$ entries in this form, but also supports loading from genetics file formats direct from disk (see next part).

```
R> CacheHaplotypes(SmallHaps)
```

The cache format is a raw binary representation that cannot be natively viewed in R. Therefore, there is a utility function, `QueryCache()`, to read all (or parts) of the cache into an R matrix representation, enabling inspection to ensure the data has loaded correctly.

For example, we can confirm that the internal cache contains `SmallHaps` by retrieving summary information. With such a small matrix we can also use `QueryCache()` to retrieve the whole matrix from the internal cache (by passing no arguments), and demonstrate that all entries match.

```
R> CacheSummary()

Cache currently loaded with 300 haplotypes, each with 400 variants.
Memory consumed: 25.60 kB.

R> all(QueryCache() == SmallHaps)

[1] TRUE
```

`QueryCache()` also supports retrieving just certain variants/haplotypes by providing a numeric vector of required variants/haplotypes respectively as the first two arguments (with standard R style 1 indexing), which is particularly useful when using **kalis** for very large problem sizes where the whole haplotype matrix is too big for R. Thus, the following code compares just the first 10 haplotypes at sites 42 and 54.

```
R> all(QueryCache(c(42, 54), 1:10) == SmallHaps[c(42, 54), 1:10])

[1] TRUE
```

Please note, it can be important to avoid mutations that appear on only a single haplotype (singletons) for numerical stability, see the Appendix (Additional file 1) for further discussion.

At this juncture we are, in principle, ready to move to discuss setting parameters and then running the LS model. However, for practical real-world problems it is rare that one would choose to load the genetic data via an R matrix, so we first discuss supported file formats.

Recommendations on loading haplotypes

With real-world large haplotype data sets it is preferable to avoid having to load them into R at all, instead having **kalis** read directly from disk into the internal optimised cache. Therefore, `CacheHaplotypes()` also supports loading from two on-disk formats directly, bypassing loading into R at all. In both cases, a string containing the file name is passed, instead of an R matrix.

Therefore, in all there are three supported methods for `CacheHaplotypes()` to load haplotype data:

- As already covered, directly from an R matrix with 0 and 1 entries. The haplotypes should be stored in columns, with variants in rows. Once loaded in the cache, the matrix can be safely removed from the R environment since it is internally stored in **kalis** in a more efficient format.
- From a `.hap.gz` file containing data in the HAP/LEGEND/SAMPLE format used by IMPUTE2 [3] and SHAPEIT [2].
- From an HDF5 file [10], with the format described in the Appendix (Additional file 1).

To facilitate transforming from data in another format, **kalis** provides `WriteHaplotypes()` and `ReadHaplotypes()` to work with the HDF5 format. This means that one can loaded genetic data into an R matrix by other means and then save into the native on-disk format recommended for **kalis**. The advantage here is that once written to disk, the R session can be restarted to eliminate the inefficient matrix representation and the haplotypes loaded directly from HDF5 to the optimised internal cache. See the Appendix (Additional file 1) for full details.

Finally, note that **kalis** will only cache and operate on one haplotype data set at a time, since the software is designed for operating at large scale. As a result, calling `CacheHaplotypes()` a second time frees the allocated cache memory and loads the new data set. Of course, there is no restriction in loading **kalis** in multiple separate R processes on the same machine, each caching different data sets.

LS model parameters

Recall that the LS model is parametrised by ρ, μ, and Π. **kalis** bundles these parameters together into an environment of class `kalisParameters`, which can be created by using the `Parameters()` function. The three key arguments to `Parameters()` are:

$\rho =$ `rho` This is a numeric vector parameter which must have length $L - 1$. Note that element `i` of this vector should be the recombination probability between variants `i` and `i+1`.

There is a utility function, `CalcRho()`, to assist with creating these recombination probabilities from a recombination map, described below.

By default, the recombination probabilities are set to zero everywhere.

$\mu =$ `mu` The mutation probabilities may be specified either as uniform across all variants (by providing a single scalar value), or may differ at each variant (by providing a vector of length L).

By default, mutation probabilities are set uniformly across variants to 10^{-8}.

$\Pi =$ `Pi` The original [6] model assumed that each haplotype has an equal prior probability of copying from any other. However, in the spirit of ChromoPainter [5] we allow a matrix of prior copying probabilities.

The copying probabilities may be specified as a standard R matrix of size $N \times N$. The element at row `j`, column `i` corresponds to the prior (background) probability that haplotype `i` copies from haplotype `j`. Note that the diagonal *must* by definition be zero and columns *must* sum to one.

Alternatively, for uniform copying probabilities, this argument need not be specified, resulting in copying probability $\frac{1}{N-1}$ between all distinct haplotypes by default.

Note 1: there is a computational cost associated with non-uniform copying probabilities, so it is recommended to leave the default of uniform probabilities when appropriate. This is achieved by omitting this argument.

Note 2: do *not* specify a uniform matrix when uniform probabilities are intended, since this would end up incurring the computational cost of non-uniform probabilities.

The `Parameters()` function accepts two further logical flag arguments, `use.speidel` (default `FALSE`) and `check.rho` (default `TRUE`). The former enables the asymmetric mutation model in RELATE [9], while the latter performs machine precision checks.

Thus, to create the default parameter set ($\rho^\ell = 0 \; \forall \ell$, $\mu^\ell = 10^{-8} \; \forall \ell$, and $\Pi_{ii} = 0 \; \forall i$, $\Pi_{ij} = (N-1)^{-1} \; \forall i \neq j$) it suffices to simply call,

```
R> pars <- Parameters()
R> pars

Parameters object with:
rho   = (0, 0, 0, ..., 0, 0, 1)
mu    = 1e-08
Pi    = 0.00334448160535117
```

Since this continues the `SmallHaps` example started above, we see $(N-1)^{-1} = (300-1)^{-1} \approx 0.003344$. In particular, note that `Parameters()` can only be invoked *after* the haplotype data

is loaded by `CacheHaplotypes()` since `Parameters()` checks that the parameter specifications are consistent with the dimensionality of the haplotype data.

Perhaps most common parameter not to leave at its default value is `rho`, since one may want to use a particular value of ρ based on a recombination map, according to Equation (3). `CalcRho()` helps with this, allowing the specification of a vector of recombination distances in centimorgans (argument `cM`), as well as the scalar multiple N_e and power γ (respectively arguments `s` and `gamma`, both defaulting to `1`), rather than having to specify ρ directly. Continuing the `SmallHaps` example, the package ships with a corresponding simulated recombination map in `SmallMap`. We can go from recombination map to recombination distances using `diff()` (and leave the default N_e and γ):

```
R> data("SmallMap")
R> rho <- CalcRho(diff(SmallMap))
R> pars <- Parameters(rho)
R> pars

Parameters object with:
rho   = (6.99999975500001e-08, 9.99999995000001e-09, 4.999999875e-08, ...,
  3.99999992000053e-08, 1.39999990200002e-07, 1)
mu    = 1e-08
Pi    = 0.00334448160535117
```

Setting up the HMMs

At this point of the analysis pipeline, the haplotypes are loaded into the internal optimised cache, and the parameters for the LS model have been defined. The final step before running the forward/backward algorithm, is to setup the storage for the N independent HMM forward and backward probabilities, $\alpha^{\ell}_{\cdot i}$ and $\beta^{\ell}_{\cdot i}, i \in \{1, \ldots, N\}$.

kalis uses $N \times N$ matrices wrapped in list objects which are of class `kalisForwardTable` and `kalisBackwardTable` respectively to store the forward/backward probabilities at a variant ℓ. These objects additionally contain which site, ℓ, the matrix represents and for performance reasons also retains the vector of N scaling constants associated with the N HMMs (corresponding to Equation (5) for the forward and Equation (6) for the backward). These objects can be created with `MakeForwardTable()` and `MakeBackwardTable()`, and at a minimum the parameters of the LS model to be used must be supplied.

Hereinafter, we refer to the collective contents of these as the 'forward table', 'backward table', or simply 'table'.

Continuing the `SmallHaps` example,

```
R> fwd <- MakeForwardTable(pars)
R> fwd

Full Forward Table object for 300 haplotypes.
Newly created table, currently uninitialised to any variant (ready for
  Forward function next).
Memory consumed: 723.98 kB

R> bck <- MakeBackwardTable(pars)
R> bck
```

```
Full Backward Table object for 300 haplotypes, in rescaled probability space.
Newly created table, currently uninitialised to any variant (ready for
  Backward function next).
Memory consumed: 724.13 kB
```

These forward and backward tables of HMMs are now ready to be 'propagated' along the genome.

Running the LS model

Everything is in place now to run the LS model. The forward eqs. (9) and (10), and backward eqs. (11) and (12), can now be executed by using the `Forward()` and `Backward()` functions respectively. These must both, as a minimum, be supplied with a corresponding forward/backward table and with the parameters of the model (in each of the first two arguments, `fwd`/`bck` respectively and `pars`). By default this will result in a single variant move either forwards or backwards. Moves directly to a designated variant can be achieved by specifying the third argument (`t`).

Note: that **kalis** seeks to minimize memory creation and copying for increased performance, and so the tables supplied to `Forward()` and `Backward()` *are modified in place.*

Continuing the `SmallHaps` example, we can see this in action, whereby the `fwd` table created in the previous subsection is propagated without assignment, first initialising to $\ell = 1$ with the first call to `Forward()`, then moving to $\ell = 2$ with the second call to `Forward()`:

```
R> Forward(fwd, pars)
R> fwd

Full Forward Table object for 300 haplotypes.
Current variant = 1
Memory consumed: 723.98 kB

R> Forward(fwd, pars)
R> fwd

Full Forward Table object for 300 haplotypes.
Current variant = 2
Memory consumed: 723.98 kB
```

Likewise we can propagate according to the backward equations:

```
R> Backward(bck, pars)
R> bck

Full Backward Table object for 300 haplotypes, in rescaled probability space.
Current variant = 400
Memory consumed: 724.13 kB

R> Backward(bck, pars)
R> bck

Full Backward Table object for 300 haplotypes, in rescaled probability space.
Current variant = 399
Memory consumed: 724.13 kB
```

In-place modification of tables

A few additional comments on the in-place modification behaviour are in order since this is contrary to the idiomatic R style, where arguments are usually unaffected by function calls. The convention in R would typically be that if an argument is to be updated, it is copied inside the function and the modified copy returned: this would incur an unacceptably high performance penalty in many intended use cases for **kalis**, hence in place modification of the tables. Indeed, for particularly large N problems, it may only be possible to hold a few $N \times N$ tables in memory at once so duplication is impossible.

This has important knock-on ramifications if the user wishes to create a duplicate of the table, due to the copy-on-write semantics of R. As stated in the R Internals documentation [7, §1.1.2]:

> "R has a 'call by value' illusion, so an assignment like `b <- a` appears to make a copy of `a` and refer to it as `b`. However, if neither `a` nor `b` are subsequently altered there is no need to copy. [...] When an object is about to be altered [...] the object must be duplicated before being changed."

The low level in-place modification by **kalis** does not alert the R memory manager, meaning that a user who executes the following will have unexpected results:

```
R> fwd2 <- fwd
R> Forward(fwd2, pars)
```

One might expect `fwd2` to now be 1 variant further advanced than `fwd`, but in fact both are references to the *same* object, so both appear to have moved a variant. For this reason, **kalis** provides the `CopyTable()` function which is the only supported method of copying the content of a table. The order of arguments in `CopyTable()` is designed to mimic assignment, with the destination on the left.

Again, in the interest of minimising functions which cause memory allocation, `CopyTable()` copies between *existing* tables. Therefore to achieve the effect intended in the previous code snippet, the correct approach is to create a destination table and then copy:

```
R> fwd2 <- MakeForwardTable(pars)
R> CopyTable(fwd2, fwd)
R> Forward(fwd2, pars)
```

After these three lines are executed, `fwd2` will be a forward table propagated one variant further than `fwd`.

Decoding a single variant

In practice, the objective of an analysis using the LS model is to propagate both a forward and backward table of N HMMs to a common variant ℓ, then compute the posterior marginal probabilities (Equation (13)) or distances (Equation (4)).

Continuing our `SmallHaps` example, let us compute both quantities at $\ell = 250$. This is now straight-forward, as we first propagate directly to the destination ℓ,

```
R> Forward(fwd, pars, 250)
R> Backward(bck, pars, 250)
R> fwd
```

```
Full Forward Table object for 300 haplotypes.
Current variant = 250
Memory consumed: 723.98 kB

R> bck

Full Backward Table object for 300 haplotypes, in rescaled probability space.
Current variant = 250
Memory consumed: 724.13 kB
```

Note that if this was a larger example, this would be an instance where the total compute time to reach $\ell = 250$ would be smaller by moving in this single function call, as opposed to making multiple calls to reach this variant.

Now that `fwd` and `bck` are at the same variant, they can be combined to obtain p^ℓ or d^ℓ, and the distance matrix plotted (see Fig.S1 for output).

```
R> p <- PostProbs(fwd, bck)
R> d <- DistMat(fwd, bck)
R> plot(d)
```

Summary

This section has carefully walked through the pipeline involved in running **kalis** for the LS model. The following code is the full pipeline brought together for readability, without asides:

```
R> library("kalis")
R> data("SmallHaps")
R> data("SmallMap")
R>
R> CacheHaplotypes(SmallHaps)
R>
R> rho <- CalcRho(diff(SmallMap))
R> pars <- Parameters(rho)
R>
R> fwd <- MakeForwardTable(pars)
R> bck <- MakeBackwardTable(pars)
R>
R> Forward(fwd, pars, 250)
R> Backward(bck, pars, 250)
R>
R> p <- PostProbs(fwd, bck)
R> d <- DistMat(fwd, bck)
```

Advanced topics

There are a couple of ‘advanced’ topics worth mentioning which are available in the **kalis** API.

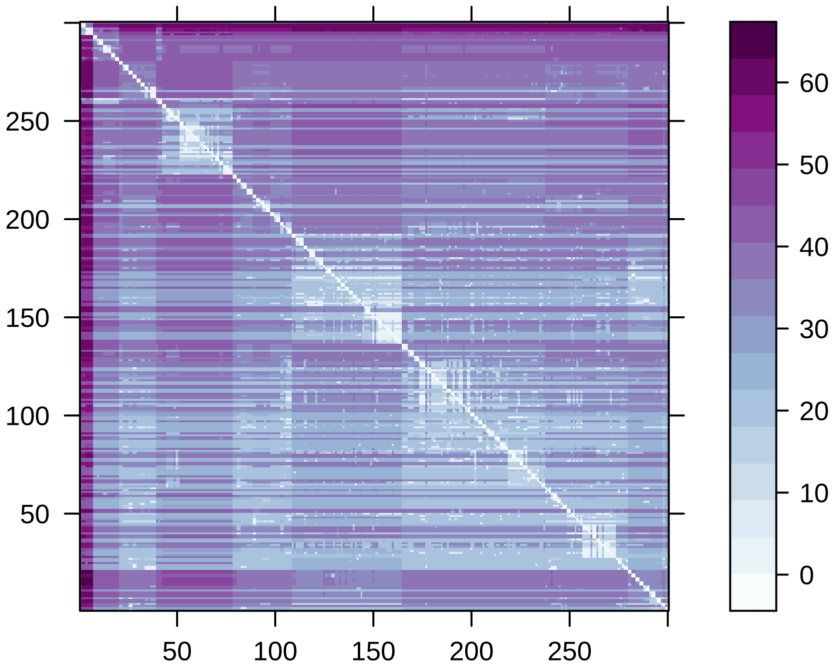

Fig. S1: Distance Matrix produced by in the `Small Haps` example.

Handling massive haplotype datasets

Firstly, in order to support massive haplotype sizes, it is possible when making a table to create only a window of recipients (ie for some subset of $i \in \{1, \dots, N\}$). The HMMs are all independent, so this facilitates propagating batches of columns of the full $N \times N$ matrix of independent HMMs on different machines without requiring network communication. By default, `MakeForwardTable()` and `MakeBackwardTable()` will include all recipients, but a range can be specified with the arguments `from_recipient` and `to_recipient`.

For example, in a problem where $N = 100,000$, a single table would require just over 80GB of memory. If one were working with a cluster where machines only have 32GB of RAM each, then one might choose to work with batches of $12,500$ recipients (ie columns) and propagate them independently on each machine, since a table then requires just over 10GB. For the forward table, this would be created by,

```
R> # Machine 1
R> fwd <- MakeForwardTable(pars, 1, 12500)
R> # Machine 2
R> fwd <- MakeForwardTable(pars, 12501, 25000)
R> # ...
R> # Machine 8
R> fwd <- MakeForwardTable(pars, 87501, 100000)
```

Likewise for the backward tables. All other operations are unaffected, from caching through to forward/backward propagation.

Notice that the posterior probabilities and distance matrices are easily computed in a distributed manner, involving only a Hadamard product and single reduction operation, so that the final results can be computed entirely distributed without having to actually form a $100,000 \times 100,000$ matrix in memory on a single machine.

Fine control of parallelism

Although the SIMD instruction set is fixed at compile time, as discussed above, the user can of course control the degree of threading at run-time.

Both `Forward()` and `Backward()` functions accept an `nthreads` argument. By default this uses the core R **parallel** package to automatically detect the number of available cores and uses all of them. However, as is common in multi-threaded packages, the user can also specify a scalar value here to use a different degree of parallelism.

There is a further option of interest to advanced users. If an integer vector is supplied, instead of a single scalar value, then **kalis** will attempt to pin threads to the corresponding core number, if the platform you are using supports thread affinity. This can be particularly useful on massive non-uniform memory access (NUMA) systems, where different cores have different speed of access to different parts of memory (cores are split over 'nodes' and while they can access all memory, it will be faster to access memory on the same node). On a Linux system, any memory allocation will by preference try to be allocated on the same node as the core. Thus, an advanced user can opt to launch as many R processes as there are NUMA nodes and use `taskset` on Linux to pin one process to one core on each NUMA node. Then, by availing of the table recipient windowing described above, these tables memory allocations will be occur on the node local to each core. Finally, when calling `Forward()/Backward()`, a vector of only the cores on the corresponding NUMA node can be provided to `nthreads`, so that essentially all cross-NUMA node memory accesses are eliminated for maximum performance.

References

[1] Csárdi, G., Hester, J., Wickham, H., Chang, W., Morgan, M., and Tenenbaum, D. *remotes: R Package Installation from Remote Repositories, Including 'GitHub'*, 2021. R package version 2.4.2.

[2] Delaneau, O., Marchini, J., and Zagury, J.-F. A linear complexity phasing method for thousands of genomes. *Nature methods 9*, 2 (2012), 179–181.

[3] Howie, B. N., Donnelly, P., and Marchini, J. A flexible and accurate genotype imputation method for the next generation of genome-wide association studies. *PLoS genetics 5*, 6 (2009).

[4] ISO. *ISO/IEC 9899:2018 Information technology — Programming languages — C*, fourth ed. BSI, June 2018.

[5] Lawson, D. J., Hellenthal, G., Myers, S., and Falush, D. Inference of population structure using dense haplotype data. *PLoS genetics 8*, 1 (2012), e1002453.

[6] Li, N., and Stephens, M. Modeling linkage disequilibrium and identifying recombination hotspots using single-nucleotide polymorphism data. *Genetics 165*, 4 (2003), 2213–2233.

[7] R Core Team. *R Internals v4.2.2.* R Foundation for Statistical Computing, Vienna, Austria, 2022.

[8] R Core Team. *R: A Language and Environment for Statistical Computing.* R Foundation for Statistical Computing, Vienna, Austria, 2023.

[9] Speidel, L., Forest, M., Shi, S., and Myers, S. R. A method for genome-wide genealogy estimation for thousands of samples. *Nature Genetics 51* (2019), 1321–1329.

[10] The HDF Group. Hierarchical Data Format, version 5, 1997-2022.
